# Respiratory syncytial virus burden in children under 2 years old in understudied areas worldwide: gap analysis of available evidence, 2012–2022

**DOI:** 10.3389/fped.2024.1452267

**Published:** 2024-11-21

**Authors:** Rodrigo Sini de Almeida, João Leite, Jessica E. Atwell, Malak Elsobky, Jorge LaRotta, Mostafa Mousa, Karan Thakkar, Mark A. Fletcher

**Affiliations:** ^1^ Vaccines and Antivirals Medical Affairs, Emerging Markets Region, Pfizer, São Paulo, Brazil; ^2^ IQVIA, Inc., Porto Salvo, Portugal; ^3^ Global Respiratory Vaccines and Antivirals, Pfizer, Collegeville, PA, United States; ^4^ Vaccines and Antivirals Medical Affairs, Emerging Markets Region, Pfizer Canada ULC, Kirkland, QC, Canada; ^5^ Latin America Vaccines Medical Affairs, Pfizer, Bogota, Colombia; ^6^ Pfizer Vaccines Medical Affairs, Riyadh, Saudi Arabia; ^7^ Emerging Asia Vaccines Medical Affairs, Pfizer, Singapore, Singapore; ^8^ Vaccines and Antivirals Medical Affairs, Emerging Markets Region, Pfizer, Paris, France

**Keywords:** respiratory syncytial virus, gap analysis, hospitalization rate, mortality, infants, lower respiratory tract infections, bronchiolitis, pneumonia

## Abstract

**Background:**

We evaluated published evidence (2012–2022) on pediatric RSV burden in 149 countries within World Health Organization (WHO) regions of Africa (AFRO), Americas (AMRO, excluding Canada and the USA), Eastern Mediterranean (EMRO), Europe (EURO, excluding European Union countries and the UK), Southeast Asia (SEARO), and Western Pacific (WPRO, excluding Australia, China, Japan, New Zealand, and South Korea).

**Methods:**

Gap analysis on RSV-associated disease (hospitalizations, hospital course, mortality or case fatality, detection, and incidence) in children ≤2 years old, where hospitalization rates, hospital course, mortality rate, case fatality rate (CFR), and postmortem detection rates were summarized, by region, for each country.

**Results:**

Forty-two publications were identified covering 19% of included countries in AFRO, 18% in AMRO, 14% in EMRO, 15% in EURO, 18% in SEARO, and 13% in WPRO. Methods, case definitions, and age groups varied widely across studies. Of these 42 publications, 25 countries reported hospitalization rate, hospital course, mortality rate, CFR, and/or postmortem detection rate. RSV hospitalization rate (per 1,000 children per year/child-years) was higher among ≤3-month-olds (range, 38 in Nicaragua to 138 in the Philippines) and ≤6-month-olds (range, 2.6 in Singapore to 70 in South Africa) than in 1–2-year-olds (from 0.7 in Guatemala to 19 in Nicaragua). Based on 11 studies, in AFRO (South Africa), AMRO (Chile and Mexico), EMRO (Lebanon and Jordan), EURO (Israel and Turkey), and SEARO (India), hospitalized children ≤2 years old remained hospitalized for 3–8 days, with 9%–30% requiring intensive care and 4%–26% needing mechanical ventilation. Based on a study in India, community-based CFR was considerably higher than that in the hospital (9.1% vs. 0% in ≤3-month-olds; 7.1% vs. 2.8% in ≤6-month-olds).

**Conclusions:**

National and regional heterogeneity of evidence limits estimates of RSV burden in ≤2-year-olds in many WHO region countries, where further country-specific epidemiology is needed to guide prioritization, implementation, and impact assessment of RSV prevention strategies.

## Introduction

The burden of neonatal and infant respiratory syncytial virus (RSV) disease is mostly due to lower respiratory tract infection (LRTI) such as bronchiolitis and pneumonia (1, 2). Although prematurity or cardiopulmonary comorbidities can increase the severity of RSV illness (3), young age remains a crucial risk factor (4). This has been well documented in the USA, for example, where a comprehensive, nationally representative analysis underscored that RSV bronchiolitis is the leading cause of hospitalization in infants <1 year old (5). Other studies in Australia, China, Japan, and New Zealand confirm the substantial burden of RSV-LRTI in the neonatal and infant period of the first 6 months of life, notwithstanding any comorbid conditions (6–9). Likewise, a European, prospective birth cohort study of children born between 2017 and 2020 documented that two-thirds of RSV-LRTI hospitalizations were among neonates and young infants (≤3 months old), and half occurred in full-term, otherwise healthy, infants (10).

Despite the substantial RSV burden literature, evidence gaps still exist worldwide, especially outside of North America, the European Union (EU), and some Western Pacific countries, likely due to challenges in establishing an infectious etiology based on incomplete surveillance or inconsistency in viral testing (11). Nonetheless, it is estimated that low-resource countries account for both >95% of global pediatric RSV-LRTI and associated deaths and 65% of global costs associated with pediatric RSV hospitalizations, posing a drain on already overwhelmed local health systems (2, 12). The World Health Organization (WHO) has emphasized the need for additional evidence from these countries to inform decision-making on RSV prevention strategies. Specifically, the WHO highlighted the importance of an availability of RSV hospitalization and mortality rates, which are often inadequately substantiated (13).

We conducted a targeted literature review (TLR) and gap analysis in countries that are typically underrepresented in the pediatric RSV-LRTI literature. We highlighted neonatal and infant hospitalization rates, hospital course, and mortality, as well as fatality and postmortem RSV positivity proportions, to underscore the burden of disease on healthcare systems and contemporary possibilities for prevention strategies.

## Methods

### Literature search

This TLR/gap analysis was conducted in accordance with guidelines from the Cochrane Handbook for Systematic Reviews of Interventions (14), the Centre for Reviews and Dissemination (University of York) (15), and PRISMA (Preferred Reporting Items for Systematic Reviews and Meta-Analyses) (16). The OvidSP interface and PICOS (patient/population, intervention, comparison, outcomes, and study) framework were used to identify English-language publications indexed in Embase and Medline between 2012 and 2022. We chose this timeframe to cover up to 11 years of existing research, incorporating more recent evidence compared to previously published global reviews (1, 2). Conference abstracts published between 2020 and (up to 29 September) 2022 were also captured within Embase.

Search strategies for the terms relevant to disease area, interventions, and study designs were developed through the combination of free-text words, indexing terms (e.g., Emtree terms for Embase), and by using Boolean terms (e.g., “and” and “or”). These relevant words and terms were reflected in the search strings (presented in Supplementary Table S1), which were appropriately modified to fit each database-specific syntax.

Abstracts and titles identified during the search were screened by a reviewer against predetermined eligibility criteria outlined in Supplementary Table S2 using the PICOS framework. Publications selected as potentially relevant were retained for full-text review. A second reviewer assessed all abstracts and publications where uncertainty existed.

Data were extracted from the selected full texts using a standardized data extraction template (MS Excel). The list of variables for extraction is outlined in Supplementary Table S3.

### Geographic coverage

The search comprised 149 countries within the WHO regions of Africa (AFRO), Americas (AMRO, excluding Canada and USA), Eastern Mediterranean (EMRO), Europe (EURO, excluding EU countries and the UK), Southeast Asia (SEARO), and Western Pacific (WPRO, excluding Australia, China, Japan, New Zealand, and South Korea) (17). The full list of included countries is presented in Table 1.

**Table 1:** List of countries included in the six WHO regions**.**

Region	Countries
African Region (AFRO): 47/47 countries included in our searches	Algeria, Angola, Benin, Botswana, Burkina Faso, Burundi, Cabo Verde, Cameroon, Central African Republic, Chad, Comoros, Congo, Cote d’Ivoire, Democratic Republic of Congo, Equatorial Guinea, Eritrea, Ethiopia, Gabon, Gambia, Ghana, Guinea, Guinea-Bissau, Kenya, Lesotho, Liberia, Madagascar, Malawi, Mali, Mauritania, Mauritius, Mozambique, Namibia, Niger, Nigeria, Rwanda, São Tomé and Príncipe, Senegal, Seychelles, Sierra Leone, South Africa, South Sudan, Swaziland, Togo, Uganda, United Republic of Tanzania, Zambia, Zimbabwe
Region of the Americas (AMRO): 33/35 countries included in our searches	Antigua and Barbuda, Argentina, Bahamas, Barbados, Belize, Bolivia, Brazil, Canada, Chile, Colombia, Costa Rica, Cuba, Dominica, Dominican Republic, Ecuador, El Salvador, Grenada, Guatemala, Guyana, Haiti, Honduras, Jamaica, Mexico, Nicaragua, Panama, Paraguay, Peru, Saint Kitts and Nevis, Saint Lucia, Saint Vincent and the Grenadines, Suriname, Trinidad and Tobago, United States, Uruguay, Venezuela
Eastern Mediterranean Region (EMRO): 22/22 countries included in our searches	Afghanistan, Bahrain, Djibouti, Egypt, Iran, Iraq, Jordan, Kuwait, Lebanon, Libya, Morocco, Oman, Pakistan, Palestine, Qatar, Saudi Arabia, Somalia, Sudan, Syria, Tunisia, United Arab Emirates, Yemen
European Region (EURO): 13/53 countries included in our searches	Albania, Andorra, Armenia, Austria, Azerbaijan, Belarus, Belgium, Bosnia and Herzegovina, Bulgaria, Croatia, Cyprus, Czech Republic, Denmark, Estonia, Finland, France, Georgia, Germany, Greece, Hungary, Iceland, Ireland, Israel, Italy, Kazakhstan, Kyrgyzstan, Latvia, Lithuania, Luxembourg, Malta, Moldova, Monaco, Montenegro, Netherlands, North Macedonia, Norway, Poland, Portugal, Romania, Russia, San Marino, Serbia, Slovakia, Slovenia, Spain, Sweden, Switzerland, Tajikistan, Turkey, Turkmenistan, Ukraine, United Kingdom, Uzbekistan
Southeast Asia Region (SEARO): 11/11 countries included in the searches	Bangladesh, Bhutan, India, Indonesia, Maldives, Myanmar, Nepal, North Korea, Sri Lanka, Thailand, Timor-Leste
Western Pacific Region (WPRO): 23/28 countries included in the searches	Australia, Brunei, Cambodia, China, Cook Islands, Fiji, Japan, Kiribati, Laos, Malaysia, Marshall Islands, Micronesia, Mongolia, Nauru, New Zealand, Niue, Palau, Papua New Guinea, Philippines, Samoa, Singapore, Solomon Islands, South Korea, Taiwan, Tonga, Tuvalu, Vanuatu, Vietnam

WHO, World Health Organization.

Grayed-out countries were not included in our searches.

### Gap analysis and RSV burden description

The burden of RSV-LRTI is specified characteristically by epidemiological parameters like detection rate, incidence, hospitalization rate, hospital course, and mortality. To better identify published data on RSV disease burden, we evaluated data availability for each of these parameters. To summarize the available evidence, we focused especially on hospitalization and death, which have been highlighted by WHO as influential for decision-making around prevention strategies (13). This evidence included RSV-associated hospitalization and mortality rates, lethality (i.e., the proportion of deaths among children with confirmed RSV infection), and postmortem RSV detection rates. The hospital course was summarized where available.

This TLR/gap analysis aimed to describe the burden of RSV regardless of risk factors; thus, we do not highlight in the body of the manuscript the few studies stratifying hospitalizations by gestational age. Nonetheless, for completeness, details are presented from these studies in pre-term children that estimated RSV-associated hospitalizations (Supplementary Table S4), hospital course (Supplementary Table S5), RSV detection rates/RSV-positive cases among hospitalized children (Supplementary Table S6), and RSV infection incidence and outpatient visit rates (Supplementary Table S7).

### Data reporting

RSV-associated hospitalization and mortality rates (as well as incidence rates in Supplementary Table S7) were standardized to 1,000 children per year, 1,000 child-years, or 1,000 live births. Fatality is described as case fatality rate (CFR) with confirmed RSV, and CFR is presented as in-hospital-based (a percentage of deaths among hospitalized children) or as community-based (a percentage of deaths among non-hospitalized children). Postmortem RSV detection rate is reported as a percentage of RSV-positive cases among all-cause or respiratory deaths. To try to systematize the substantial variability in age group categories applied across studies, we grouped the age range used by each investigator within one of four strata: ≤3 months, ≤6 months, ≤1 year, or ≤2 years.

Across all outcomes, data are reported with one decimal place for numbers <10 and rounded to the nearest whole number for values 10 or greater.

## Results

### Literature search outcomes and study characteristics

**Figure 1:**
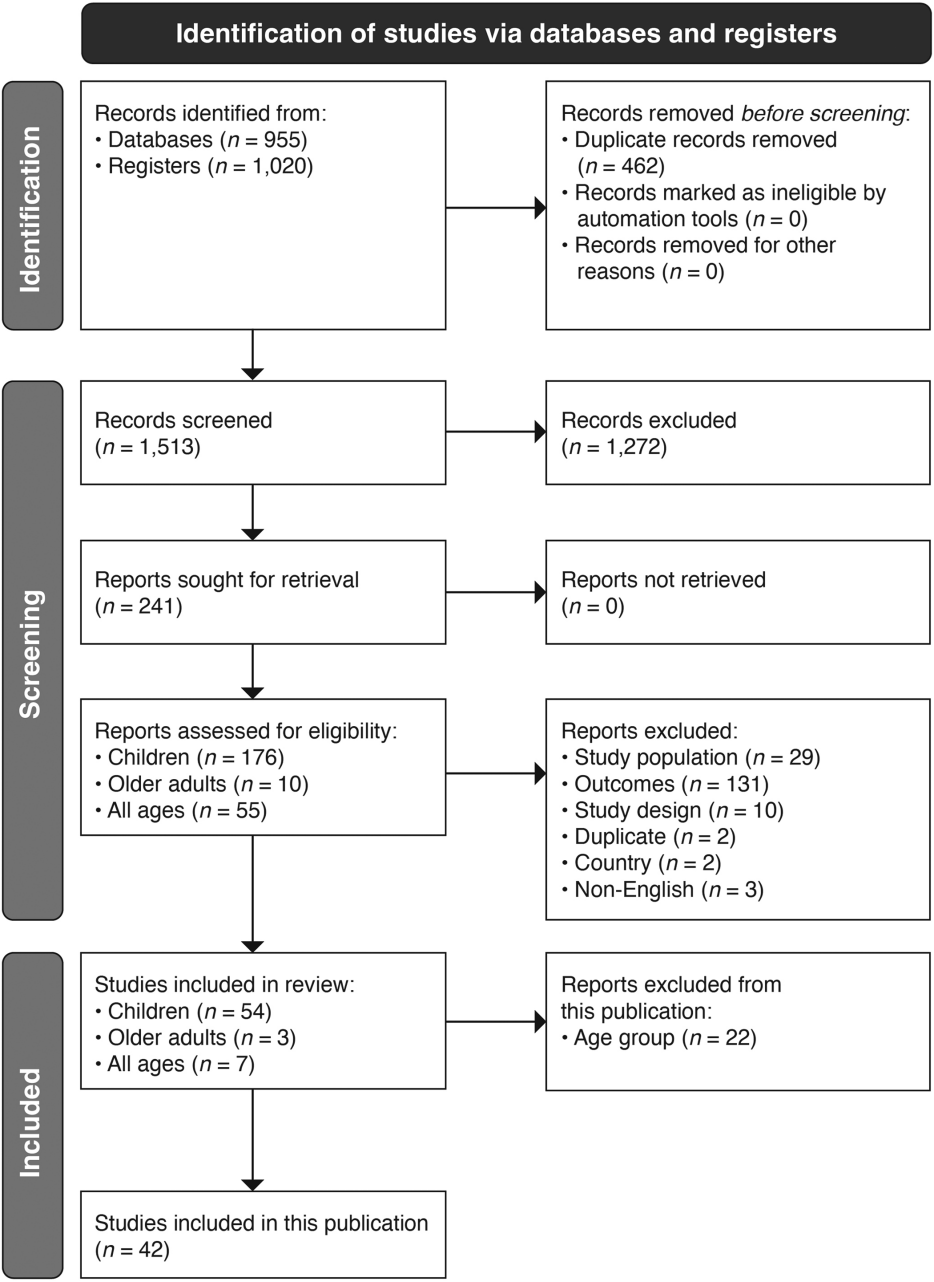
PRISMA flow diagram for study identification, screening, and inclusion.

Of the 1,975 references screened, 42 publications (18–59) that reported at least incidence, hospitalization rate, hospital course, detection rate, or mortality were selected (Figure 1). Sample sizes varied greatly, from 16 neonates admitted to the neonatal intensive care unit (ICU) with acute respiratory infection (ARI) in India (37) to 530,345 children hospitalized for all respiratory/circulatory causes including pneumonia and influenza in South Africa (43). Community data were available in 29% (12/42) of studies. Study designs also varied, encompassing “surveillance” (16 studies), “cross-sectional design” (5 studies), “retrospective” cohort (5 studies), “prospective” cohort (12 studies), both “retrospective and prospective” cohort (1 study), or statistical modeling (3 studies). Clinical case definitions for RSV positivity spanned from ARI (12 studies), lower respiratory tract infections (ranging from LRTI in general to specific clinical presentations like pneumonia and bronchiolitis, 19 studies) to fever-based definitions [influenza-like infection (ILI) or severe ARI (SARI), 7 studies], or a general definition of “symptomatic illness” (4 studies). RSV detection was based exclusively on polymerase chain reaction (PCR) in more than half of the studies (60%, 25/42). Other detection methods included immunoassays (14%, 6/42) and antigen tests (14%, 6/42). Some studies incorporated more than one method (i.e., some of the study samples were analyzed with one method and others used another method), specifically immunofluorescence and PCR (4.8%, 2/42) or antigen test and PCR (2.4%, 1/42). The detection method was not reported in two studies. More details on the study characteristics are described in Supplementary Table S8.

### GAP analysis

The 42 publications identified in this study covered 17% (25/149) of the targeted countries (18–59). By WHO region, this represented 19% (9/47) of countries in AFRO, 18% (6/33) in AMRO, 14% (3/22) in EMRO, 15% (2/13) in EURO, 18% (2/11) in SEARO, and 13% (3/23) in WPRO.

**Figure 2:**
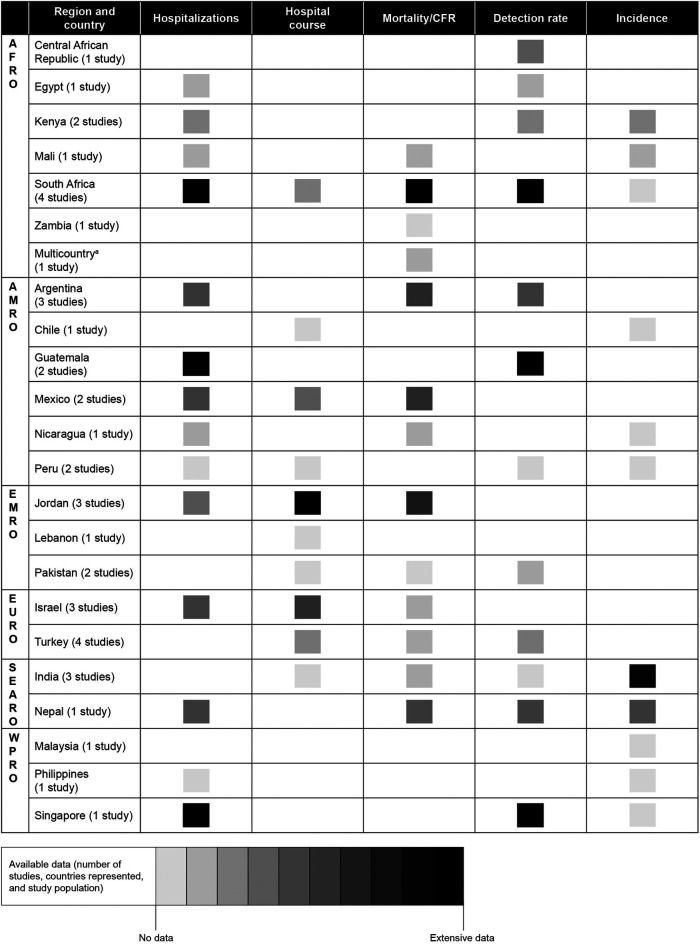
Data availability for children ≤2 years old in countries of the WHO regions of AFRO, AMRO (except Canada and USA), EMRO, EURO (except for European Union countries and the UK), SEARO, and WPRO (excluding Australia, China, Japan, New Zealand, and South Korea). AFRO, African Region; AMRO, Region of the Americas; EMRO, Eastern Mediterranean Region; EURO, European Region; SEARO, Southeast Asia Region; WPRO, Western Pacific Region. ^a^Ethiopia, Kenya, Mali, Mozambique, Sierra Leone, and South Africa. Country coverage within each region: AFRO, 19% (9 of 47 countries); AMRO 18% (6 of 33 countries); EMRO, 14% (3 of 22 countries); EURO, 15% (2 of 13 countries); SEARO 18% (2 of 11 countries); WPRO 13% (3 of 23 countries).

Details on the availability of evidence on RSV-associated hospitalization rate, hospital course, RSV-associated mortality/CFR, RSV viral detection rate, and incidence of RSV disease are presented by WHO regions/countries in Figure 2.

In AFRO, 5 of 11 studies were conducted in South Africa (23, 26, 27, 43, 59). The most available information in South Africa pertained to RSV-associated hospitalization, mortality or CFR, and viral detection rates, while hospital course and incidence rates were scarce. From other countries in AFRO beyond South Africa, only sparse information was available on hospitalizations (Egypt, Kenya, and Mali) (24, 28, 49), mortality or CFR (Ethiopia, Kenya, Mali, Mozambique, Sierra Leone, and Zambia) (23, 24, 31), viral detection rates (Central African Republic, Egypt, and Kenya) (28, 41, 49), and disease incidence (Kenya and Mali) (24, 47).

In AMRO, 7 of 11 studies in this region were concentrated in Argentina, Guatemala, and Mexico (21, 22, 29, 30, 33, 45, 56). Most evidence primarily pertained to RSV-associated hospitalizations and viral detection rates from Guatemala (30, 45), although Argentina and Mexico reported hospitalization, mortality or CFR, and viral detection rates (21, 22, 29, 33, 56). Only limited evidence was available on hospital course and incidence of RSV disease in this region, based on sparse information from Chile, Mexico, Nicaragua, and Peru (42, 44, 56, 57).

In EMRO, evidence came mainly from Jordan, with 3 of 6 studies conducted in this country (32, 40, 58). The most substantial Jordan data pertained to hospitalizations, hospital course, and mortality or CFR, while Lebanon and Pakistan provided limited information on hospital course, mortality, and viral detection rates (18, 20, 39). Notably, evidence on the incidence of RSV disease was completely lacking in EMRO.

In EURO, data were only available from Israel and Turkey (seven studies in total) (19, 34–36, 38, 48, 52), with most evidence from Israel that pertained to hospitalization rates and hospital course (36, 38). Only limited data were available for mortality and viral detection rates in these countries (19, 36, 48). As with EMRO, evidence on the incidence of RSV disease was completely lacking in EURO.

In SEARO, India and Nepal provided incidence estimations (25, 50), while some evidence from a single study in Nepal was available for hospitalizations, mortality or CFR, and viral detection rates (25). Limited data were available in India on hospital course (37).

In WPRO, Singapore provided clear results about hospitalization and viral detection rates (53), and there was some information on disease incidence from Singapore, as well as Malaysia and the Philippines (53–55). Hospital course and mortality were unavailable from this region.

In the following sections of this manuscript, we summarize data—available from 31 studies covering 25 countries across the six WHO regions—for RSV-associated hospitalization rates and hospital course, mortality rates, and CFR.

### Hospitalization rates and hospital course

Hospitalization rates per 1,000 children per year or per child-years are presented in Table 2 and hospital course in Table 3.

**Table 2:** RSV-associated hospitalization rates per 1,000 children per year/child-years grouped by upper age-range categories ≤3 months, ≤6 months, ≤12 months, and ≤24 months (with the age group as reported by the investigators).

Hospitalization rates per 1,000 children per year/child-years by age group								
Region	Country	Population	Clinical presentation	≤3 months	≤6 months	≤12 months	≤24 months	Ref.
AFRO	Egypt	Children within Damanhur district hospitalized at facilities with respiratory illness	ARI^b,d^	–	–	17 (1–11)	–	(49)
	Kenya	Children residing in Karemo Division and enrolled in the surveillance system	SARI^a^	–	13 (0–5)	14 (6–11)	8.1 (12–23)	(28)
	Mali	Children of women within an influenza RCT who develop ILI or pneumonia and are RSV tested	ILI or pneumonia^b^	–	46 (0–6)	–	–	(24)
	South Africa	Children born from women enrolled in an influenza clinical trial, followed up for 2 years, who develop LRTI during follow-up	LRTI^b,c^	–	70 (0–6)	50 (0–12)	10 (13–24)	(59)
							30 (0–24)	
	South Africa	Children in age group with private health insurance in South Africa who attend the private hospitals under study	Pneumonia or influenza^b,d^	–	–	31 (0–11)	–	(43)
			All-respiratory^b,d^	–	–	76 (0–11)	–	
AMRO	Argentina	Estimated annual children population in the catchment area of Buenos Aires	SARI^a^	–	–	24 (0–11)	14 (0–23)	(29)
	Argentina	Estimated annual children population in the catchment area of Buenos Aires	Severe LRTI^a^	–	–	30 (0–11)	–	(33)
	Guatemala	Children in Santa Rosa and Quetzaltenango populations who seek care at surveillance sites	ARI^a^	–	8.7–65^f^ (0–5)	3.2–27^f^ (6–11)	0.7–4.8^f^ (12–23)	(30)
						5.9–46^f^ (0–11)		
	Guatemala	Children within the census populations of Santa Rosa and Quetzaltenango	ARI^a^	–	21 (0–5)	–	5.1 (6–23)	(45)
	Nicaragua	Children followed up since birth within the Nicaraguan Influenza Birth Cohort Study	Symptomatic illness and reported or measured fever^b^	38 (0–2)	4.8 (3–5)	33 (6–11)	19 (12–23)	(42)
							23 (0–23)	
EMRO	Jordan	Children residing in the city of Amman and attending Al-Bashir Hospital	Fever and/or respiratory symptoms^e^	–	21–26^g^ (0–5)	6.0–8.0^g^ (6–11)	2.0–3.0^g^ (12–23)	(40)
							8.0–9.0^g^ (0–23)	
EURO	Israel	Children in the estimated population served by a medical center in Hadera sub-district	LRTI^e^	–	–	–	5.7 (0–23)	(38)
	Turkey	Children attending hospitals for LRTI in Turkey	LRTI^a^	–	–	13 (0–11)	7.7 (0–24)	(35)
			Acute bronchiolitis^a^	–	–	7.8 (0–11)	4.6 (0–24)	
			Pneumonia^a^	–	–	5.6 (0–11)	3.2 (0–24)	
SEARO	Nepal	Infants of women enrolled in an RCT followed up from birth	ARI^b^	–	2.7 (0–6)	–	–	(25)
WPRO	Philippines	Children attending healthcare facilities in 2 municipalities on main Biliran Province island	ARI^b^	138 (0–1)	21 (2–5)	12 (6–11)	14 (12–23)	(55)
	Singapore	Children seeking care at all hospitals in Singapore (statistical model)	LRTI^b^	–	34 (0–5)	–	13 (6–29)	(53)
			Bronchiolitis^b^	–	30 (0–5)	–	9.9 (6–29)	
			Pneumonia^b^	–	2.6 (0–5)	–	2.4 (6–29)	
			Complicated pneumonia^b^	–	0.7 (0–5)	–	0.8 (6–29)	

AFRO, African Region; AMRO, Region of the Americas (except Canada and USA); ARI, acute respiratory infection; EMRO, Eastern Mediterranean Region; EURO, European Region (excluding EU countries and the UK); ILI, influenza-like infection; LRTI, lower respiratory tract infection; mo, months; RCT, randomized controlled trial; Ref., reference; RSV, respiratory syncytial virus; SARI, severe acute respiratory infection; SEARO, Southeast Asia Region; WPRO, Western Pacific Region (except Australia, China, Japan, New Zealand, and South Korea).

All rates were recalculated to 1,000 children per year/child-years, unless otherwise specified. Numbers <10 are reported with one decimal digit; numbers ≥10 have been rounded to a whole number.

^a^ Hospitalizations per 1,000 children per year.

^b^ Hospitalizations per 1,000 child-years.

^c^ Recalculated from one child-year.

^d^ Recalculated from 100,000 child-years.

^e^ Hospitalizations per 1,000 children.

^f^ Ranges between sites and years.

^g^ Range across years.

**Table 3:** Summary of information on hospital course available from the reviewed studies**.**

Region	Country	Population	Age, months	Hospital, days	Mechanical ventilation	ICU admission	ICU, days	Ref.
AFRO	South Africa	Out/inpatients children with RSV bronchiolitis		Mean			Mean (range)	(27)
			0–24	6.7	–	30%	9.5 (1–32)	
AMRO	Chile	Infants admitted to hospital with confirmed RSV-LRTI		Mean (SE)				(44)
			0–5	2.4 (0.4) Mild, 4.3 (0.3) Moderate, 11 (4) Severe	22%	24%	–	
	Mexico	Infants admitted to hospital with confirmed RSV-LRTI	0–5	–	–	8.1%	–	(56)
			6–12	–	–	3.8%	–	
			12–24	–	–	0.5%	–	
EMRO	Jordan	Inpatient children with RSV and fever and/or respiratory symptoms		Median (IQR)				(32)
			0–23	4 (3–7)	5.9%	9.8%	–	
	Jordan	Inpatient children with RSV and fever and/or respiratory symptoms					Median (IQR)	(40)
			0–23	–	4.0%	9.0%	5 (3–7)	
	Jordan	Inpatient children with RSV and fever and/or respiratory symptoms		Mean				(58)
			0–23	5	4.1%	9.0%	–	
	Lebanon	Infants admitted to hospital with respiratory symptoms and confirmed RSV	0–1	–	7.1%	71%	–	(18)
			1–6	–	5.3%	29%	–	
EURO	Israel	Children admitted to ICU with RSV bronchiolitis		Median (IQR)			Median (IQR)	(36)
			0–24	8 (8)	26%	–	4 (5)	
	Israel	Children hospitalized with RSV bronchiolitis		Median (range)				(38)
			0–23	3 (1–34)	–	–	–	
	Turkey	Infants admitted to ICU with RSV-LRTI					Median (range)	(19)
			0–5	–	–	–	8 (2–180)	
SEARO	India	Newborns admitted to ICU with RSV-ARI	0–1	–	17%	–	Median (IQR)	(37)
							8 (5–14)	

AFRO, African Region; AMRO, Region of the Americas (except Canada and USA); ARI, acute respiratory infection; EMRO, Eastern Mediterranean Region; EURO, European Region (excluding EU countries and the UK); ICU, intensive care unit; IQR, interquartile range; LRTI, lower respiratory tract infection; mo, months; Ref., reference; RSV, respiratory syncytial virus; SE, standard error; SEARO, Southeast Asia Region.

Numbers <10 are reported with one decimal digit; numbers ≥10 have been rounded to a whole number.

In AFRO, hospitalization details were available from six studies in four countries for the age groups 0–6 months and 1–2 years, specifically Egypt, Kenya, Mali, and South Africa (24, 27, 28, 43, 49, 59). Conversely, there was no information specific to newborns ≤3 months old.

Age-specific hospitalization rates decreased with older age. Among young infants 0–6 months old, the rates ranged from 13 per 1,000 child-years in Kenya (28) to 70 per 1,000 child-years in South Africa (59); among older infants 6–11 months old, the rate was 14 per 1,000 children per year in Kenya (28); and among children 1–2 years old, the rate ranged from 8.1 per 1,000 children per year in Kenya (28) to 10 per 1,000 child-years in South Africa (59). In South Africa, children ≤2 years old with RSV-LRTI spent on average 7 days in the hospital, and 30% of them were admitted to the ICU with an average stay of 9–10 days (27).

In AMRO, there were seven studies in five countries across the age ranges of 0–2 months to 1–2 years, specifically from Argentina, Chile, Guatemala, Mexico, and Nicaragua (29, 30, 33, 42, 44, 45, 56). Hospitalization rates among neonates aged 0–2 months old were 38 per 1,000 child-years in Nicaragua (42); among infants 0–5 months old, rates ranged from 8.7 to 65 per 1,000 children per year in Guatemala (30); among older infants aged 6–11 months, from 3.2 per 1,000 children per year in Guatemala (30) to 33 per 1,000 child-years in Nicaragua (42); and among children 1–2 years old, from 0.7 per 1,000 children per year in Guatemala (30) to 19 per 1,000 child-years in Nicaragua (42). In two studies, one each in Chile and Mexico, documenting hospital course among infants 0–5 months old, the average length of stay varied by RSV-LRTI severity, from 2 days for mild illness to 4 days for moderate illness and 11 days for severe illness; ICU admission rates were 8%–24%, with 22% requiring mechanical ventilation (44, 56).

In EMRO, hospitalization data were available from four studies in two countries, specifically Jordan and Lebanon (18, 32, 40, 58). Hospitalization rates were only available in Jordan, with the age ranges of 0–5 months to 1–2 years: among young infants 0–5 months old, the rates were 21–26 per 1,000 children per year; among older infants, the rates for 6–11 months were 6–8 per 1,000 children per year, and among children 1–2 years old, the rates were 2–3 per 1,000 children per year (40). Average hospital stay duration, available only for infants 0–23 months old in Jordan, was 4–5 days (32, 58), while in Lebanon, ICU admission rates were 71% for neonates (0–1 month old), 29% for infants (1–6 months old), and 13% for children (6–24 months old) (18).

In EURO, hospitalization details were available in children—although not specifically for the ≤6-month-old age group—from two studies in Turkey (19, 35) and two studies in Israel (36, 38). RSV-LRTI hospitalization rate among infants 0–11 months old in Turkey was 13 per 1,000 children per year (35), while in children ≤2 years old values ranged from 6 per 1,000 children per year in Israel (38) to 8 per 1,000 children per year in Turkey (35). In RSV-LRTI-hospitalized children ≤2 years old, the average hospital stay was 3 to 8 days in Israel, the average ICU stay was from 4 days in Israel to 8 days in Turkey, and of the children in Israel admitted to the ICU, 27% required mechanical ventilation (19, 36, 38).

In SEARO, hospitalization rates were available from one study in Nepal (25), and there was hospital course information from one study in India (37). The Nepal study reported a 2.7 per 1,000 child-years RSV-ARI hospitalization rate in young infants ≤6 months old (25). In India, 17% of neonates (0–1 month old) admitted to the ICU with RSV-ARI required mechanical ventilation with an average length of ICU stay of 8 days (37).

In WPRO, hospitalization was investigated in one study from Singapore (53) and one in the Philippines (55). In Singapore, the hospitalization rates were 34 per 1,000 child-years in infants 0–5 months old and 13 per 1,000 child-years in infants and children 6–29 months old (53). The Philippines study focused on neonates 0–1 month old, reporting an RSV-ARI hospitalization rate of 138 per 1,000 child-years, the highest documented across all countries and regions (55). No data on hospital course were available from WPRO.

### Mortality rates

Mortality rates per 1,000 child-years or children per year (Table 4) were available from five studies in four countries, specifically one study in AFRO (South Africa) (26), three studies in AMRO (Argentina and Nicaragua) (29, 33, 42), and one study in SEARO (India) (51). In AMRO, in infants 0–12 months old, the mortality rate in Argentina was 0.3 per 1,000 child-years in the hospital (29) and ranged between 0.7 per 1,000 child-years and 0.9 per 1,000 live births when hospital and community deaths were combined (29, 33). Among children 0–23 months old, the community-based mortality rate was 2.8–4.2 per 1,000 child-years in Nicaragua (42). In SEARO, only the study in India had data for the ≤6-month-old age group, with combined community and hospital mortality rates per 1,000 child-years of 3.0 for newborns 0–3 months old, 2.5 for young infants 0–6 months old, 1.6 for infants 0–12 months old, and 1.0 for children 0–23 months old (51). The authors also compared community and hospital mortality rates in India and found that the mortality rates per 1,000 child-years, by age group, were substantially greater in the community as compared with the hospital: 3.0 vs. 0 (0–3 months old); 2.3 vs. 0.3 (0–6 months old); 1.5 vs. 0.1 (0–12 months old); and 0.9 vs. 0.1 (0–23 months old) (51).

**Table 4:** RSV-associated mortality rates per 1,000 children per year/child-years, grouped by upper age-range categories ≤3 months, ≤6 months, ≤12 months, and ≤24 months (with the age group as reported by the investigators).

Region	Country	Population	Rate definition	Setting	≤3 months	≤6 months	≤12 months	≤24 months	Ref.
AFRO	South Africa	Children within the population of South Africa	Excess RSV-associated all-cause, respiratory, circulatory deaths per 1,000 child-years^a^	Hospital and community	–	–	1.4 (0–11)	–	(26)
AMRO	Argentina	All in-hospital or community ARI deaths	RSV-SARI associated deaths per 1,000 children per year	Hospital	–	–	0.3 (0–12)	–	(29)
				Hospital and community	–	–	0.7 (0–12)	–	
	Argentina	Children born in the catchment area of Buenos Aires	Severe RSV-LRTI-associated deaths per 1,000 live births	Hospital and community	–	–	0.9 (0–11)	–	(33)
	Nicaragua	Children in the Nicaraguan Influenza Birth Cohort Study	RSV-LRTI-associated deaths per 1,000 child-years	Community	–	–	–	2.8–4.2^b^ (0–23)	(42)
SEARO	India	Children from 93 villages in Melghat, or visiting the 5 primary health centers, or hospitalized at 3 hospitals of the villages	RSV-LRTI-associated deaths per 1,000 child-years	Hospital	0.0 (0–3)	0.3 (0–6)	0.1 (0–12)	0.1 (0–23)	(51)
				Community	3.0 (0–3)	2.3 (0–6)	1.5 (0–12)	0.9 (0–23)	
				Hospital and community	3.0 (0–3)	2.5 (0–6)	1.6 (0–12)	1.0 (0–23)	

AFRO, African Region; AMRO, Region of the Americas (except Canada and USA); LRTI, lower respiratory tract infection; mo, months; Ref., reference; RSV, respiratory syncytial virus; SARI, severe acute respiratory infection; SEARO, Southeast Asia Region.

All rates were recalculated to 1,000 children per year/child-years, unless otherwise specified. Numbers <10 are reported with one decimal digit; numbers ≥10 have been rounded to a whole number.

^a^ Recalculated from 100,000 child-years.

^b^ Range between deaths that occurred within 14 days and 46 days from onset of symptoms.

### Case fatality rate (CFR)

CFR, in-hospital (Table 5) or community-based (Table 6), was documented among 13 studies in nine countries: specifically, 2 studies in AFRO (Mali and South Africa) (24, 27); 4 studies in AMRO (Argentina and Mexico) (21, 29, 33, 56); 3 studies in EMRO (Jordan) (32, 40, 58); 2 studies in EURO (Israel and Turkey) (19, 36); and 2 studies in SEARO (India and Nepal) (25, 51). Only the study in India documented CFR in newborns 0–3 months old (51), and only three studies from Mali (24), India (51), and Nepal (25) reported community CFR.

**Table 5:** RSV-associated in-hospital CFR grouped by upper age-range categories ≤3 months, ≤6 months, ≤12 months and ≤24 months (with the age group as reported by the investigators)**.**

Proportion (%) of deaths among children hospitalized with RSV infection (in-hospital CFR) by age group							
Region	Country	Denominator	≤3 months	≤6 months	≤12 months	≤24 months	Ref.
AFRO	South Africa	Children (outpatient or inpatient) with proven RSV bronchiolitis	–	–	–	4.9% (0–24)	(27)
AMRO	Argentina	Children hospitalized with RSV-LRTI	–	–	–	0.5% (0–23)	(21)
	Argentina	Children hospitalized with RSV-SARI	–	–	–	1.1%^a^ (0–23)	(29)
	Argentina	Children hospitalized with severe RSV-LRTI	–	–	0.9% (0–11)	–	(33)
	Mexico	Children hospitalized with RSV-LRTI	–	1.5% (0–5)	0.8% (6–11)	0.5% (12–23)	(56)
EMRO	Jordan	Children hospitalized with respiratory symptoms and/or fever due to RSV	–	–	–	0.5% (0–23)	(32)
	Jordan	Children hospitalized with respiratory symptoms and/or fever due to RSV	–	–	–	0.7% (0–23)	(40)
	Jordan	Children hospitalized with respiratory symptoms and/or fever due to RSV	–	–	–	0.5% (0–23)	(58)
EURO	Israel	Children admitted to ICU with RSV bronchiolitis	–	–	–	1.4% (0–24)	(36)
	Turkey	Infants admitted to ICU with RSV-LRTI	–	1.2% (0–5)	–	–	(19)
SEARO	India	Children hospitalized with severe LRTI	0.0% (0–3)	2.8% (0–6)	1.4% (0–12)	1.0% (0–23)	(51)
		Children hospitalized with very severe LRTI	0.0% (0–3)	0.0% (0–6)	0.0% (0–12)	0.0% (0–23)	

AFRO, African Region; AMRO, Region of the Americas (except Canada and USA); ARI, acute respiratory infection; CFR, case fatality rate; EMRO, Eastern Mediterranean Region; EURO, European Region (excluding EU countries and the UK); ICU, intensive care unit; LRTI, lower respiratory tract infection; mo, months; Ref., reference; RSV, respiratory syncytial virus; SARI, severe acute respiratory infection; SEARO, Southeast Asia Region.

Numbers <10 are reported with one decimal digit; numbers ≥10 have been rounded to a whole number.

^a^ All deaths were in children less than 1 year old.

**Table 6:** RSV-associated community-based CFR grouped by upper age-range categories ≤3 months, ≤6 months, ≤12 months, and ≤24 months (with the age group as reported by the investigators)**.**

Proportion (%) of deaths among non-hospitalized children with RSV infection (community-based CFR) by age group							
Region	Country	Denominator	≤3 months	≤6 months	≤12 months	≤24 months	Ref.
AFRO	Mali	Children of women in an influenza RCT followed up at home who develop RSV-ILI/pneumonia	–	0.7% (0–6)	–	–	(24)
SEARO	India	Children in the community with severe RSV-LRTI	9.1% (0–3)	7.1% (0–6)	2.4% (0–12)	2.4% (0–23)	(51)
		Children in the community with very severe RSV-LRTI	29% (0–3)	18% (0–6)	12% (0–12)	9.9% (0–23)	
	Nepal	Children born from women enrolled in an influenza clinical trial followed up at home who develop RSV-ARI	–	0.3% (0–6)	–	–	(25)

AFRO, African Region; ARI, acute respiratory infection; CFR, case fatality rate; ILI, influenza-like infection; LRTI, lower respiratory tract infection; mo, months; RCT, randomized controlled trial; Ref., reference; RSV, respiratory syncytial virus; SEARO, Southeast Asia Region.

Numbers <10 are reported with one decimal digit; numbers ≥10 have been rounded to a whole number.

In AFRO, age-specific CFR (RSV-ILI and RSV-pneumonia deaths) was 0.7% among ≤6-month-old infants in the community, followed within a maternal influenza vaccination trial, in Mali (24), and 4.9% (RSV bronchiolitis deaths) in children ≤2 years old from both in-hospital and outpatient settings in South Africa (27). In AMRO, in-hospital CFR was 1.5% in young infants 0–5 months old (Mexico), 0.9% in infants 0–11 months old (Argentina), and 0.5% in children 1–2 years old (Mexico) (33, 56). In EMRO, CFR was available only in the hospital setting from Jordan and ranged between 0.5% and 0.7% among infants 0–23 months old (32, 40, 58). In EMRO, CFRs were reported among infants and children admitted to the ICU: 1.2% among infants 0–5 months old in Turkey and 1.4% among children 0–24 months old in Israel (19, 36). In SEARO, the infant CFR in India associated with severe RSV-LRTI was substantially lower in the hospital than in the community: 0% vs. 9.1% among neonates and young infants 0–3 months old and 2.8% vs. 7.1% among infants 0–6 months old (51).

### Postmortem RSV detection

Four AFRO studies covering seven countries (Ethiopia, Kenya, Mali, Mozambique, Sierra Leone, South Africa, and Zambia) (23, 31), one AMRO study in Argentina (33), and one EMRO study in Pakistan (39) evaluated the proportion of RSV-positive test results among infants who died from any cause in the hospital or the community (Table 7).

**Table 7:** Postmortem RSV positivity by upper age-range categories ≤3 months, ≤6 months, ≤12 months, and ≤24 months (with the age group as reported by the investigators)**.**

Proportion (%) of deaths with RSV-positive test among all-cause deaths (postmortem RSV detection)							
Region	Country	Denominator	≤3 months	≤6 months	≤12 months	≤24 months	Ref.
AFRO	Ethiopia, Kenya, Mali, Mozambique, Sierra Leone, South Africa	Children who died at health facilities	–	6.5% (1–5)	–	–	(23)
	Zambia	Children who died at University Teaching Hospital in Lusaka	–	6.1% (0–5)	–	–	(31)
AMRO	Argentina	Infants who died at the hospital in Buenos Aires	0.5% (0–1)	–	–	16% (1–23)	(33)
		Infants who died in the community in Buenos Aires	4.8% (0–1)	–	–	16% (1–23)	
EMRO	Pakistan	Infants who died in the community in Karachi	–	3.7% (0–5)	–	–	(39)

AFRO, African Region; AMRO, Region of the Americas (except Canada and USA); EMRO, Eastern Mediterranean Region; mo, months; Ref., reference; RSV, respiratory syncytial virus.

Numbers <10 are reported with one decimal digit; numbers ≥10 have been rounded to a whole number.

Only the study in Argentina reported data for neonates 0–1 month old and children 1–23 months old comparing hospital and community deaths (33). The authors found that while the 0.5% proportion of RSV-related hospital deaths in neonates was significantly lower than the 16% in children, in the community the rate increased to 4.8% in neonates whereas it remained at 16% in children (33). All other studies reported data for infants ≤6 months old (23, 31, 39). In the AFRO countries, the proportion ranged between 6.1% among hospital deaths and 6.5% among community deaths (23, 31). In Pakistan, the RSV-related proportion was 3.7% among community deaths (39).

## Discussion

This gap analysis is based on the understanding that policymakers require national-level estimates to inform decisions regarding the introduction of prevention strategies for RSV and to assess their subsequent effects (60, 61). We reviewed the evidence published between 2012 and 2022 available on RSV burden in 149 countries across six WHO regions, excluding some countries in AMRO (Canada and USA), EURO (the EU countries and the UK), and WPRO (Australia, China, Japan, New Zealand, and South Korea). Only 17% (25 of 149) of the investigated countries had published details in children ≤2 years old when considering hospitalization rate, hospital course, mortality rate, CFR, and postmortem detection rates, as well as viral detection rate, disease incidence, and outpatient visit rates. This suggests that national evidence is limited to one-quarter of the countries considered in this WHO region gap analysis and is unavailable from more than three-quarters of the possible countries. The WHO supports evidence-based RSV prevention strategies in understudied countries, particularly identifying RSV hospitalization and death rates as crucial parameters for decision-making (13).

Based on the results of this review, evidence for these outcomes remains incomplete. First, RSV-associated hospitalization and mortality (fatality) statistics were available from only 11% of the 149 countries among children (≤24 months old), although there were few epidemiology studies among neonates and young infants (≤3 months old) who are most often severely affected by RSV-LRTI (5–10), with one study in Nicaragua and one in the Philippines reporting hospitalization rates and one study in India reporting death rates (42, 51, 55).

RSV hospitalization rates in this TLR/gap analysis for neonates and young infants (≤3 months old) ranged from 38 to 138 per 1,000 child-years. With the limited data, ranges varied widely by WHO region, and as suggested by global estimates (1, 2) they were consistently higher in younger infants (0–6 months old) than in children (1–2 years old): AFRO, 13 per 1,000 children per year to 70 per 1,000 child-years vs. 8.1 per 1,000 children per year to 10 per 1,000 child-years; AMRO, 8.7–65 per 1,000 children per year vs. 0.7 per 1,000 children per year to 19 per 1,000 child-years; EMRO, 21–26 vs. 2.0–3.0 per 1,000 children per year; and WPRO, 2.6–34 vs. 2.4–14 per 1,000 child-years. (No data for infants ≤6 months old were available in EURO, and no data in children 1–2 years old were available in SEARO.) Based on 11 hospitalization course studies in eight countries (AFRO, South Africa; AMRO, Chile, Mexico; EMRO, Jordan, Lebanon; EURO, Israel, Turkey; and SEARO, India), admitted children ≤2 years old remained in the hospital for 3–8 days, with 8%–30% of them requiring ICU admission and 4%–26% needing mechanical ventilation. Although not investigated in this TLR/gap analysis, the cost to treat and manage each severe RSV episode in low- and middle-income countries has been estimated to be $4,114, with a cost of illness accounting for almost 40% of household monthly income (62). Taken together, these data highlight the great burden on healthcare systems and on families alike, which is particularly challenging in resource-deprived countries.

Studies in EU and the USA reported that RSV-associated deaths among hospitalized infants are infrequent and can span from a value of 0 deaths in one active surveillance study covering five countries between 2017 and 2019 in Europe to 6.9 per 1,000,000 live births in a national statistics analysis in the USA between 1999 and 2018 (10, 63). In contrast, previous global systematic reviews and meta-analyses reported that there is a higher burden associated with RSV deaths in other parts of the world (1, 2, 64), like this TLR/gap analysis shows, specifically from published studies in Argentina, India, and South Africa. In neonates and infants ≤3 months old, India reported a combined hospital and community-based mortality rate associated with RSV-LRTI of 3 per 1,000 child-years (51). Most of these deaths occurred in the community with a hospital-based CFR of 0% but a community-based CFR of 9% for severe and 29% for very severe cases (51). In infants ≤1 year old, mortality rates ranged from 0.1 per 1,000 child-years (India) to 0.3 per 1,000 children per year (Argentina) in the hospital setting and from 0.7 per 1,000 children per year (Argentina) to 1.6 per 1,000 child-years (India) when community deaths were also considered (29, 51). CFR was up to 12% in India in the community among infants ≤1 year old (51) and up to 4.8% in South Africa in the hospital among children ≤2 years old (27). These RSV-associated death statistics likely reflect lower access to care and availability of resources in more vulnerable healthcare systems, as compared to the values reported from the EU and USA (2, 64).

Despite the RSV-LRTI burden that can vary by calendar year across geographies, the substantial differences between countries revealed by this TLR/gap analysis could also be attributable to methodological inconsistencies (61, 65). Research settings (e.g., hospital vs. community or inpatient vs. outpatient) can contribute to the variability, as well as can access to care that varies across economies (2, 27). Inconsistency in case definitions also contributes to data heterogeneity. For example, case definitions based on the requirement of fever, such as for ILI and SARI, might underestimate the RSV burden because many clinical cases present without fever (66, 67). Some methods of sample collection and viral detection have higher sensitivity and specificity than others, and some patient sampling sites are more suited for identifying certain viruses (68). Altogether, this highlights the urgent need for standardized protocols as a foundation for surveillance.

One of our TLR's key strengths is that it encompasses evidence from 149 countries worldwide that historically have been understudied. Over an 11-year publication period (2012–2022), we assembled results reported in these studies across five possible clinical outcomes to identify and highlight gaps in the RSV burden epidemiology for children aged ≤2 years within countries across all six WHO regions. As with all TLRs, missing relevant publications, due to English-language and period-of publication restrictions, represent inherent limitations.

## Conclusions

The WHO has long identified RSV-LRTI as a major cause of infant morbidity and mortality, but the existing evidence is still sparse and varies considerably both among countries and within regions. Our gap analysis—conducted outside North America, the EU, and a few Western Pacific nations—could find published evidence for only 13%–19% of the investigated countries within the WHO regions. When considering age groups, this TLR/gap analysis underscored missing evidence across all WHO regions in the neonate and infant age groups (≤3 and ≤6 months of age), even though they are most susceptible to RSV disease across all clinical outcomes and severities. Most of the reported outcomes pertained to hospitalizations, mortality or CFR, and viral detection rates, as available from AFRO (South Africa), AMRO (Argentina, Guatemala, and Mexico), EMRO (Jordan), EURO (Israel and Turkey), SEARO (India), and WPRO (Singapore). In contrast, information on hospital course and disease incidence was scant across regions. In addition to an underlying scarcity of published results, limitations in the study methodologies as well as a substantial variability in the reported outcomes might result both in an underestimation of burden and in challenges to any cross-study comparisons between countries or across WHO regions. To facilitate impact assessment of prevention strategies, research based on well-established methods and standardized protocols is needed to enhance the accuracy of national disease burden estimates.
