# Supplementary material for: Respiratory syncytial virus burden in children under 2 years old in understudied areas worldwide: gap analysis of available evidence, 2012–2022
Source: Front Pediatr. 2024 Nov 21;12:1452267. doi: 10.3389/fped.2024.1452267 (PMC11617186; doi:10.3389/fped.2024.1452267)
Supplement: Supplementary file 1 [file Table1.pdf]

Supplementary Table S1. Web-search strategy for RSV infection

No.	Embase® search strategy
1	exp Human respiratory syncytial virus/ or exp respiratory syncytial virus infection/ or exp Respiratory Syncytial Virus Infections/ or exp Respiratory Syncytial Virus, Human/ or exp Respiratory Syncytial Viruses/ or (respiratory syncytial virus$ or human respiratory syncytial virus$ or rsv or hrsv).ti,ab.
2	exp infant/ or exp newborn/ or (infan* or newborn* or new born or new borns or newly born or neonat* or baby* or babies or premature or prematures or prematurity or preterm* or pre term or premies or low birth weight or low birthweight or VLBW or LBW or ELBW or NICU).ti,ab,kw.
3	exp maternal age/ or (maternal or pregnancy or gestational or prenatal).ab,ti,kw.
4	(latin america or South America or Central America or (LATAM or (Belize or Costa Rica or El Salvador or Guatemala or Honduras or Mexico or Nicaragua or Panama or Argentina or Bolivia or Brazil or Chile or Colombia or Ecuador or French Guiana or Guyana or Paraguay or Peru or Suriname or Uruguay or Venezuela or Cuba or Dominican Republic or Haiti))).mp.
5	APAC.mp. or Asia/ or Asia pacific.mp. or (Afghanistan or American Samoa or Armenia or Azerbaijan or Bangladesh or Bhutan or Brunei Darussalam or Cambodia or Cook Islands or Democratic People's Republic of Korea or Fiji or French Polynesia or Georgia or Guam or Hong Kong, or India or Indonesia or Iran or Kazakhstan or Kiribati or Kyrgyzstan or Lao People's Democratic Republic or Macao, or Malaysia or Maldives or Marshall Islands or Micronesia or Mongolia or Myanmar or Nauru or Nepal or New Caledonia or Niue or Northern Mariana Islands or Pakistan or Palau or Papua New Guinea or Philippines or Samoa or Singapore or Solomon Islands or Sri Lanka or Tajikistan or Thailand or Timor-Leste or Tonga or Turkey or Turkmenistan or Tuvalu or Uzbekistan or Vanuatu or Vietnam).mp.
6	"Africa south of the Sahara"/ or South Africa/ or Africa/ or North Africa/ or Central Africa/ or Africa.mp. or middle east.mp. or Middle East/ or (Afghanistan or Algeria or Ancient Near East or Angola or Bahrain or Benin or Botswana or Burkina Faso or Burundi or Cameroon or Cape Verde or Central African Republic or Chad or Comoros or Congo or Cyprus or Djibouti or Egypt or Equatorial Guinea or Eritrea or Ethiopia or Gabon or Gambia or Ghana or Guinea or Iran or Iraq or Israel or Jordan or Kenya or Kuwait or Lebanon or Lesotho or Liberia or Libya or Madagascar or Malawi or Mali or Mauritania or Mauritius or Morocco or Mozambique or Namibia or Niger or Nigeria or Oman or Palestine or Qatar or Rwanda or Saudi Arabia or Senegal or Seychelles or Sierra Leone or Somalia or South Africa

	or South Sudan or Sudan or Swaziland or Syria or Tanzania or Togo or Tunisia or Uganda or United Arab Emirates or UAE or Western Sahara or Yemen or Zambia or Zimbabwe).mp.
7	(Armenia or Azerbaijan or Belarus or Georgia or Kazakhstan or Kyrgyzstan or Moldova or the Russian Federation or Tajikistan or Turkey or Turkmenistan or Ukraine or Uzbekistan).mp.
8	4 or 5 or 6 or 7
9	(animal$ not human$).mp. or (animal/ not (animal/ and human/)) or (animal/ or animal experiment/ or animal model/ or animal tissue/ or nonhuman/) or (news or comment or editorial or letter or note or case reports).pt. or (letter/ or historical article/ or case report/ or editorial/)
10	(1 and 2 and 8) not 9
11	(1 and 3 and 8) not 9
12	10 or 11
13	(1 and 8) not 9
14	limit 10 to yr="2012 -Current"
15	limit 11 to yr="2012 -Current"
16	14 or 15
17	limit 13 to yr="2012 -Current"
**No.**	**MEDLINE® search strategy**
1	exp Human respiratory syncytial virus/ or exp respiratory syncytial virus infection/ or exp Respiratory Syncytial Virus Infections/ or exp Respiratory Syncytial Virus, Human/ or exp Respiratory Syncytial Viruses/ or (respiratory syncytial virus$ or human respiratory syncytial virus$ or rsv or hrsv).ti,ab.
2	exp infant/ or exp newborn/ or (infan* or newborn* or new born or new borns or newly born or neonat* or baby* or babies or premature or prematures or prematurity or preterm* or pre term or premies or low birth weight or low birthweight or VLBW or LBW or ELBW or NICU).ti,ab,kw.
3	exp maternal age/ or (maternal or pregnancy or gestational or prenatal).ab,ti,kw.

4	(latin america or South America or Central America or (LATAM or (Belize or Costa Rica or El Salvador or Guatemala or Honduras or Mexico or Nicaragua or Panama or Argentina or Bolivia or Brazil or Chile or Colombia or Ecuador or French Guiana or Guyana or Paraguay or Peru or Suriname or Uruguay or Venezuela or Cuba or Dominican Republic or Haiti))).mp.
5	APAC.mp. or Asia/ or Asia pacific.mp. or (Afghanistan or American Samoa or Armenia or Azerbaijan or Bangladesh or Bhutan or Brunei Darussalam or Cambodia or Cook Islands or Democratic People's Republic of Korea or Fiji or French Polynesia or Georgia or Guam or Hong Kong, or India or Indonesia or Iran or Kazakhstan or Kiribati or Kyrgyzstan or Lao People's Democratic Republic or Macao, or Malaysia or Maldives or Marshall Islands or Micronesia or Mongolia or Myanmar or Nauru or Nepal or New Caledonia or Niue or Northern Mariana Islands or Pakistan or Palau or Papua New Guinea or Philippines or Samoa or Singapore or Solomon Islands or Sri Lanka or Tajikistan or Thailand or Timor-Leste or Tonga or Turkey or Turkmenistan or Tuvalu or Uzbekistan or Vanuatu or Vietnam).mp.
6	"Africa south of the Sahara"/ or South Africa/ or Africa/ or North Africa/ or Central Africa/ or Africa.mp. or middle east.mp. or Middle East/ or (Afghanistan or Algeria or Ancient Near East or Angola or Bahrain or Benin or Botswana or Burkina Faso or Burundi or Cameroon or Cape Verde or Central African Republic or Chad or Comoros or Congo or Cyprus or Djibouti or Egypt or Equatorial Guinea or Eritrea or Ethiopia or Gabon or Gambia or Ghana or Guinea or Iran or Iraq or Israel or Jordan or Kenya or Kuwait or Lebanon or Lesotho or Liberia or Libya or Madagascar or Malawi or Mali or Mauritania or Mauritius or Morocco or Mozambique or Namibia or Niger or Nigeria or Oman or Palestine or Qatar or Rwanda or Saudi Arabia or Senegal or Seychelles or Sierra Leone or Somalia or South Africa or South Sudan or Sudan or Swaziland or Syria or Tanzania or Togo or Tunisia or Uganda or United Arab Emirates or UAE or Western Sahara or Yemen or Zambia or Zimbabwe).mp.
7	(Armenia or Azerbaijan or Belarus or Georgia or Kazakhstan or Kyrgyzstan or Moldova or the Russian Federation or Tajikistan or Turkey or Turkmenistan or Ukraine or Uzbekistan).mp.
8	4 or 5 or 6
9	(animal$ not human$).mp. or (animal/ not (animal/ and human/)) or (animal/ or animal experiment/ or animal model/ or animal tissue/ or nonhuman/) or (news or comment or editorial or letter or note or case reports).pt. or (letter/ or historical article/ or case report/ or editorial/)
10	(1 and 2 and 8) not 9
11	(1 and 3 and 8) not 9

12	10 or 11
13	(1 and 8) not 9
14	limit 10 to yr="2012 -Current"
15	limit 11 to yr="2012 -Current"
16	14 or 15
17	limit 13 to yr="2012 -Current"

Supplementary Table S2. Eligibility criteria for article selection

PICOS	Inclusion criteria	Exclusion criteria
Population	• RSV infection among the maternal immunization population<br>○ ≤2 years old	• Patients without RSV<br>• Children >2 years old<br>• RSV infection during influenza (H1N1) pandemic
Intervention/comparator	• No restriction	• NA
Outcomes	• Epidemiology:<br>○ Incidence<br>○ Prevalence<br>○ Mortality/fatality rate<br>• Any testing and diagnosis methods of RSV<br>• Clinical and humanistic burden<br>○ Hospitalization<br>○ Hospital course<br>○ HRQoL; sequelae<br>○ Complications due to RSV	• Any outcomes not listed in the inclusion criteria<br>• Caregiver burden<br>• Genetic testing/profiling<br>• Economic burden

**PICOS**	**Inclusion criteria**	**Exclusion criteria**
Study design(s)	• Any observational studies designed to measure relevant outcomes (epidemiological, clinical, and humanistic burden) including cohort studies, cross-sectional studies, longitudinal (prospective/retrospective) studies, case-control studies, systematic reviews, etc.	• Animal studies<br>• *In vitro* studies<br>• Editorials, notes, comments<br>• Case studies<br>• Simple reviews
Geographic limits (based on WHO regions)	• Africa Region (AFRO)<br>• Regions of the Americas (AMRO, except for the USA and Canada)<br>• Eastern Mediterranean Region (EMRO)<br>• European Region (EURO) to include Armenia, Azerbaijan, Belarus, Georgia, Kazakhstan, Kyrgyzstan, Moldova, the Russian Federation, Tajikistan, Turkey, Turkmenistan, Ukraine, Uzbekistan (except for European Union countries)<br>• Southeast Asia Region (SEARO)<br>• Western Pacific Region (WPRO, except for China, Australia, Japan, South Korea, and New Zealand)	• All other countries not listed in the inclusion criteria
Other limits	• Time frame: 2012 to date<br>• English-language papers	

HRQoL, health-related quality of life; NA, not applicable; PICOS, patient/population, intervention, comparison, outcomes, and study; RSV, respiratory syncytial virus; WHO, World Health Organization.

Supplementary Table S3. List of variables for data extraction

**Criterion**	**Variable**
Study characteristics	• Publication details (author, year, title, journal/proceeding)<br>• Study design<br>• Study population
Patient characteristics	• Patient population (pediatric population)<br>• Gender<br>• Ethnicity<br>• Age: mean/median (SD/SE/range/IQR)<br>• RSV: upper respiratory tract disease/lower respiratory tract disease<br>• RSV strain (RSV-A or RSV-B)<br>• Duration of disease: mean (SD)<br>• Treatment<br>• Comorbidities
Outcomes	• Incidence<br>• Prevalence<br>• Mortality/lethality

Criterion	Variable
	• Impact of COVID-19 on epidemiology of RSV<br>• Seasonal and cyclical trends<br>• Testing and diagnosis<br>• Clinical and humanistic burden<br>○ Patient-focused hospitalization<br>○ HRQoL<br>○ RSV-associated mortality<br>○ Complications due to RSV<br>○ Emergency department visits<br>Variation by population subgroups (for all outcomes where data are available)

HRQoL, health-related quality of life; IQR, interquartile range; RSV, respiratory syncytial virus; SD, standard deviation; SE, standard error.

Supplementary Table S4. RSV-associated hospitalizations per 1,000 children per year/child-years by gestational age

**Study and population**	**Rate definition**	**Upper age range (actual age group)**	
		**≤12 mo**	**≤24 mo**
AMRO			
Mexico – Newborns resident in San Luis Potosí, Soledad de Graciano Sánchez, or Mexquitic de Carmona municipalities and admitted to 2 local hospitals (22)	RSV-ARI 1,000 child-years in pre-term children (<37 weeks' GA)	63–94<br>(0–12 mo)	–
Peru – Newborns born at 3 hospitals in Lima or transferred to these hospitals (46)	RSV-ARI per 1,000 child-years in pre-term children (weight ≤1500 g)	116<br>(0–12 mo)	–
EURO			
Israel – Infants admitted to the SUMC pediatric emergency room, pediatric wards, and pediatric ICU diagnosed with alveolar pneumonia (34)	RSV-CAAP per 1,000 children in full-term children (>36 weeks' GA)<br>RSV-CAAP per 1,000 children in pre-term children (31–36 weeks' GA)	–	5.8<br>(0–23 mo)<br>15<br>(0–23 mo)

AMRO, Region of the Americas; ARI, acute respiratory infection; CAAP, community-acquired alveolar pneumonia; EURO, European Region; GA, gestational age; ICU, intensive-care unit; mo, months; RSV, respiratory syncytial virus; SUMC, Soroka University Medical Center.
Note: Numbers <10 are reported with one decimal digit; numbers ≥10 have been rounded to a whole number.

Supplementary Table S5. Hospital course by gestational age

Study and population	Age group	Gestational age	ICU admission rate	Mechanical ventilation rate	Mean days in the hospital (SD)
AMRO					
Peru – Newborns born at or transferred to 3 hospitals in Lima diagnosed with ARI (46)	0–12 mo	Pre-term infants (weight ≤ 1500 g)	–	–	8.2 (9.3)
EURO					
Israel – Infants admitted to the SUMC pediatric ER, pediatric wards, and pediatric ICU diagnosed with alveolar pneumonia (34)	0–23 mo	Pre-term (31–36 weeks' GA)<br><br>Full-term (>36 weeks' GA)	11%<br><br>2.9%	–	–
Turkey – Children presenting at ER or outpatient departments of 15 hospitals across Turkey and admitted with LRTI (48)	0–24 mo	Pre-term (29–35 weeks' GA)	23%	–	9.5 (8.2)

AMRO, Region of the Americas; ARI, acute respiratory infection; ER, emergency room; EURO, European Region; GA, gestational age; ICU, intensive-care unit; LRTI, lower respiratory tract infection; mo, months; SD, standard deviation; SUMC, Soroka University Medical Center.
Note: Numbers <10 are reported with one decimal digit; numbers ≥10 have been rounded to a whole number.

Supplementary Table S6. RSV detection rates

Country	Denominator	Numerator	Upper age range (actual age group)			
			**≤3 mo**	**≤6 mo**	**≤12 mo**	**≤24 mo**
**RSV-positive cases among hospitalized children (%)**						
**Studies in all children irrespective of their gestational age**						
AFRO						
Kenya (28)	Children hospitalized with SARI or presenting at visits with ILI	RSV-SARI		13% (0–5 mo)	12% (6–11 mo)	12% (12–23 mo)
		RSV-ILI		2.8% (0–5 mo)	11% (6–11 mo)	9.4% (12–23 mo)
Central African Republic (41)	Children presenting with ILI and those hospitalized with SARI at sentinel sites	RSV-SARI/ILI		13% (0–6 mo)	7.5% (7–12 mo)	6.0% (13–24 mo)
South Africa (27)	In-/outpatient children with proven viral bronchiolitis	RSV-bronchiolitis				14% (0–24 mo)
South Africa (43)		RSV-LRTI			35% (0–11 mo)	

	Children hospitalized for all respiratory/circulatory causes including pneumonia and influenza	RSV-ARI			36%<br>(0–11 mo)	
Egypt (49)	Hospitalized patients with ARI	RSV-ARI			51%<br>(1–11 mo)	
AMRO						
Argentina (21)	Children hospitalized with LRTI	RSV-LRTI				53%<br>(0–23 mo)
Argentina (29)	Children hospitalized with SARI	RSV-SARI				62%<br>(0–23 mo)
Argentina (33)	Children hospitalized with severe LRTI	Severe RSV-LRTI			66%<br>(0–11 mo)	
Chile (44)	Infants admitted to hospital with LRTI	RSV-LRTI		56%<br>(0–5 mo)		
Guatemala (45)	Children hospitalized with ARI	RSV-ARI		42%<br>(0–5 mo)		31%<br>(6–23 mo)
EMRO						

Jordan (32)	Children admitted to hospital with fever and/or respiratory symptoms	RSV-fever with/without respiratory symptoms				64%<br><br>(0–23 mo)
Jordan (40)	Children admitted to hospital with fever and/or respiratory symptoms	RSV-fever with/without respiratory symptoms				44%<br><br>(0–23 mo)
Jordan (58)	Children admitted to hospital with fever and/or respiratory symptoms	RSV-fever with/without respiratory symptoms				44%<br><br>(0–23 mo)
Pakistan (20)	Children hospitalized with ARI	RSV-ARI	29%<br><br>(0–2 mo)	23%<br><br>(3–5 mo)	18%<br><br>(6–11 mo)	
Lebanon (18)	Children hospitalized with respiratory symptoms	RSV-respiratory symptoms	40%<br><br>(0–1 mo)	32%<br><br>(1–6 mo)		20%<br><br>(6–24 mo)
EURO						
Israel (38)	Hospitalized children with bronchiolitis	RSV-bronchiolitis				84%<br><br>(0–23 mo)
Turkey (19)	Infants admitted to ICU with LRTI	RSV-LRTI		20%<br><br>(0–5 mo)		

Turkey (35)	Children hospitalized with LRTI	RSV-LRTI	48%<br>(0–3 mo)	37%<br>(4–6 mo)	32%<br>(7–11 mo)	25%<br>(12–24 mo)<br>38%<br>(0–24 mo)
Turkey (52)	Children hospitalized with respiratory failure due to LRTI	RSV-LRTI				17%<br>(0–23 mo)
SEARO						
India (37)	Newborns admitted to ICU with ARI	RSV-ARI	88%[a]<br>(0–1 mo)			
WPRO						
Malaysia (54)	Children hospitalized with LRTI	RSV-LRTI				23%<br>(1–24 mo)
Singapore (53)	Children hospitalized with LRTI (bronchiolitis and pneumonia)	RSV-LRTI		47%<br>(0–5 mo)		34%<br>(6–29 mo)<br>42%<br>(0–29 mo)

**Studies in pre-term children**						
AMRO						
Mexico (22)	Pre-term neonates (<37 weeks' GA) hospitalized with ARI	RSV-ARI			36%<br>(0–12 mo)	
Peru (46)	Pre-term infants (weight ≤1500 g) with ARI requiring hospitalization or ER consultations	RSV-ARI hospitalization<br><br>RSV-ARI ER admissions			46%<br>(0–12 mo)<br><br>27%<br>(0–12 mo)	
EURO						
Turkey (48)	Premature children (29–35 weeks' GA) admitted to ER with LRTI	RSV-LRTI				34%<br>(0–24 mo)
**RSV-positive cases among outpatient visits (%)**						
**Studies in all children irrespective of their gestational age**						
AFRO						
Kenya (28)	Children presenting at clinics with ILI	RSV-ILI		2.8%<br>(0–5 mo)	11%<br>(6–11 mo)	9.4%<br>(12–23 mo)

Egypt (49)	Children presenting at clinics with ILI	RSV-ILI			4% (1–11 mo)	
AMRO						
Guatemala (45)	Children attending visits with ARI	RSV-ARI		12% (0–5 mo)		16% (6–23 mo)
**RSV-positive cases in the community (%)**						
**Studies in all children irrespective of their gestational age**						
AFRO						
South Africa (59)	Children of women enrolled during second trimester of pregnancy who develop LRTI	RSV-LRTI				21% (0–24 mo)
AMRO						
Nicaragua (42)	Infants ≤4 weeks of age at enrollment attending study visits with LRTI or symptomatic respiratory illness	RSV-LRTI<br>RSV-symptomatic illness				19% (0–23 mo)<br>35% (0–23 mo)
SEARO						

Nepal (25)	Infants with ARI born from women enrolled in influenza clinical trials and followed up at home	RSV-ARI		8.9% (0–6 mo)		
WPRO						
Philippines (55)	Children followed up at households for ARI	RSV-LRTI		16% (2–5 mo)	9% (6–11 mo)	38% (12–23 mo)
		Severe RSV-LRTI		14% (2–5 mo)	15% (6–11 mo)	50% (12–23 mo)
		Very severe RSV-LRTI		21% (2–5 mo)	28% (6–11 mo)	41% (12–23 mo)
		RSV-ARI		13% (2–5 mo)	17% (6–11 mo)	19% (12–23 mo)

AFRO, African Region; AMRO, Region of the Americas; ARI, acute respiratory infection; EMRO, Eastern Mediterranean Region; ER, emergency room; EURO, European Region; GA, gestational age; ICU, intensive-care unit; ILI, influenza-like infection; LRTI, lower respiratory tract infection; mo, months; RSV, respiratory syncytial virus; SARI, severe acute respiratory infection; SEARO, Southeast Asia; WPRO, Western Pacific Region.

Note: Numbers <10 are reported with one decimal digit; numbers ≥10 have been rounded to a whole number.

[a]86% of these were pre-term newborns.

Supplementary Table S7. RSV infection incidence and outpatient visit rates per 1000 children per year/child-years

Study and population	Rate definition	≤3 mo	≤6 mo	≤12 mo	≤24 mo
**Estimated crude incidence of infection**					
**Studies in all children irrespective of their gestational age**					
AFRO					
Kenya – Children presenting with ARI at the outpatient department of KDH hospital in Kilifi Town, Kilifi District (47)	RSV-LRTI per 1,000 child-years[a]<br>RSV-ARI per 1,000 child-years[a]	–	–	5.6 (0–11 mo)<br>14 (0–11 mo)	–
South Africa – Newborns of women enrolled in an influenza clinical trial and developed LRTI during the 2 years of follow-up (59)	RSV-LRTI per 1,000 child-years[b]	–	150 (0–6 mo)	120 (0–12 mo)	80 (0–24 mo)<br>40 (13–24 mo)
Mali – Children born from women enrolled in an influenza clinical trial followed up at home (24)	RSV-ILI or pneumonia per 1,000 child-years	–	537 (0–6 mo)	–	–
AMRO					

Study and population	Rate definition	≤3 mo	≤6 mo	≤12 mo	≤24 mo
Nicaragua – Children followed up since birth within the Nicaraguan Influenza Birth Cohort Study (42)	RSV-LRTI per 1,000 child-years	50<br>(0–2 mo)	100<br>(3–5 mo)	182<br>(6–11 mo)	109<br>(12–23 mo)<br>120<br>(0–23 mo)
	Severe RSV-LRTI per 1,000 child-years	31<br>(0–2 mo)	5<br>(3–5 mo)	22<br>(6–11 mo)	10<br>(12–23 mo)<br>15<br>(0–23 mo)
	RSV-pneumonia per 1,000 child-years	38<br>(0–2 mo)	43<br>(3–5 mo)	114<br>(6–11 mo)	63<br>(12–23 mo)<br>71<br>(0–23 mo)
	RSV-symptomatic illness with reported/measured fever per 1,000 child-years	67<br>(0–2 mo)	178<br>(3–5 mo)	361<br>(6–11 mo)	249<br>(12–23 mo)<br>248<br>(0–23 mo)

Study and population	Rate definition	≤3 mo	≤6 mo	≤12 mo	≤24 mo
Peru – Children with ARI resident in highland communities in San Marcos, Cajamarca, for ≤1 year after enrollment (57)	RSV-ARI per 1,000 child-years[c]	–	340<br>(0–5 mo)	340<br>(6–11 mo)<br>340<br>(0–12 mo)	310<br>(12–23 mo)
EMRO					
Nepal – Infants born from women enrolled in an influenza trial and followed up from birth (25)	RSV-ARI per 1,000 child-years	107<br>(1 mo)<br>246<br>(2 mo)<br>222<br>(3 mo)	256<br>(4 mo)<br>220<br>(5 mo)<br>160<br>(6 mo)	–	–
WPRO					
Philippines – Children attending healthcare facilities in 2 municipalities on a main island of Biliran province (55)	RSV-ARI per 1,000 child-years<br><br>RSV-LRTI per 1,000 child-years	224<br>(0–1 mo)<br><br>–	248<br>(2–5 mo)<br><br>124<br>(2–5 mo)	131<br>(6–11 mo)<br><br>40<br>(6–11 mo)	146<br>(12–23 mo)<br><br>74<br>(12–23 mo)

Study and population	Rate definition	≤3 mo	≤6 mo	≤12 mo	≤24 mo
	Severe RSV-LRTI per 1,000 child-years	138<br>(0–1 mo)	41<br>(2–5 mo)	26<br>(6–11 mo)	38<br>(12–23 mo)
	Very severe RSV-LRTI per 1,000 child-years	–	25<br>(2–5 mo)	19<br>(6–11 mo)	13<br>(12–23 mo)
**Studies in term and pre-term children separately**					
AMRO					
Peru – Newborns born at or transferred to 3 hospitals in Lima diagnosed with ARI (46)	RSV-ARI per 1,000 child-years in pre-term children (weight ≤1500 g)	132<br>(0–1 mo)	337<br>(2–5 mo)	178<br>(6–9 mo)<br>50<br>(10–12 mo)	–
SEARO					
India – Children in community and hospitalized with severe LRTI from 93 tribal villages in Melghat (50)	Severe RSV-LRTI per 1,000 child-years in full-term children in the community (≥37 weeks' GA)	13<br>(0–3 mo)	16<br>(0–6 mo)	19<br>(0–9 mo)<br>22<br>(0–11 mo)	21<br>(0–18 mo)<br>21<br>(0–24 mo)

Study and population	Rate definition	≤3 mo	≤6 mo	≤12 mo	≤24 mo
	Severe RSV-LRTI per 1,000 child-years in pre-term children in the community (<37 weeks' GA)	16<br>(0–3 mo)	26<br>(0–6 mo)	22<br>(0–9 mo)<br>26<br>(0–11 mo)	20<br>(0–18 mo)<br>19<br>(0–24 mo)
	Severe RSV-LRTI per 1,000 child-years in hospitalized full-term children (≥37 weeks' GA)	13<br>(0–3 mo)	13<br>(0–6 mo)	13<br>(0–9 mo)<br>14<br>(0–12 mo)	13<br>(0–18 mo)<br>12<br>(0–24 mo)
	Severe RSV-LRTI per 1,000 child-years in hospitalized pre-term children (<37 weeks' GA)	12<br>(0–3 mo)	10<br>(0–6 mo)	11<br>(0–9 mo)<br>13<br>(0–12 mo)	12<br>(0–18 mo)<br>12<br>(0–24 mo)
	Overall severe RSV-LRTI per 1,000 child-years in full-term children (≥37 weeks' GA)	15<br>(0–3 mo)	19<br>(0–6 mo)	22<br>(0–9 mo)<br>25<br>(0–12 mo)	24<br>(0–18 mo)<br>23<br>(0–24 mo)

Study and population	Rate definition	≤3 mo	≤6 mo	≤12 mo	≤24 mo
	Overall severe RSV-LRTI per 1,000 child-years in pre-term children (<37 weeks' GA)	20<br>(0–3 mo)	22<br>(0–6 mo)	19<br>(0–9 mo)<br>27<br>(0–12 mo)	22<br>(0–18 mo)<br>20<br>(0–24 mo)
	Overall very severe RSV-LRTI per 1,000 child-years in full-term children (≥37 weeks' GA)	11<br>(0–3 mo)	10<br>(0–6 mo)	9.6<br>(0–9 mo)<br>12<br>(0–12 mo)	10<br>(0–18 mo)<br>10<br>(0–24 mo)
	Overall very severe RSV-LRTI per 1,000 child-years in pre-term children (<37 weeks' GA)	7.9<br>(0–3 mo)	14<br>(0–6 mo)	14<br>(0–9 mo)<br>12<br>(0–12 mo)	10<br>(0–18 mo)<br>11<br>(0–24 mo)
Nepal – Infants born from women enrolled in an influenza trial and followed up from birth (25)	RSV-ARI per 1,000 child-years in full-term infants (≥37 weeks' GA)		551<br>(0–6 mo)		
	RSV-ARI per 1,000 child-years in pre-term infants (28–31 weeks' GA)		195<br>(0–6 mo)		

Study and population	Rate definition	≤3 mo	≤6 mo	≤12 mo	≤24 mo
**Estimated RSV-associated outpatient visit rate**					
**Studies in all children irrespective of their gestational age**					
AFRO					
Kenya – Children residing in Karemo Division and enrolled in the surveillance system (28)	RSV-ILI per 1,000 children per year		6.5<br>(0–5 mo)	41<br>(6–11 mo)	29<br>(12–23 mo)
AMRO					
Guatemala – Children within the census populations of Santa Rosa and Quetzaltenango (45)	RSV-ARI per 1,000 children per year		6.5<br>(0–5 mo)		19<br>(6–23 mo)
WPRO					
Singapore – Children seeking care at all hospitals in Singapore (statistical model) (53)	RSV-LRTI per 1,000 child-years		171<br>(0–6 mo)		69<br>(6–29 mo)

AFRO, African Region; AMRO, Region of the Americas; ARI, acute respiratory infection; EMRO, Eastern Mediterranean Region; GA, gestational age; ICU, intensive-care unit; ILI, influenza-like infection; LRTI, lower respiratory tract infection; mo, months; RSV, respiratory syncytial virus; SEARO, Southeast Asia Region; WPRO, Western Pacific Region.

Note: All rates were recalculated to 1,000 children per year/child-years, unless otherwise specified. Numbers <10 are reported with one decimal digit; numbers ≥10 have been rounded to a whole number.

[a]Recalculated from 100,000 child-years.

[b]Recalculated from one child-year.

[c]Recalculated from 100 child-years.

Supplementary Table S8. Description of the studies included in the literature review and details of their cohorts

Study	Country	Years	Study design	Setting	N	Cohort age (mo)	RSV test	Specimen
**Studies including data on children up to 2 years old, irrespective of their gestational age**								
AFRO								
Komoyo 2021 (41)	Central African Republic	Jan 2015 to Dec 2018	Surveillance	Hospital and outpatient	3,903	0–60	PCR	Nasopharyngeal swab
Rowlinson 2013 (49)	Egypt	Apr 2009 to Mar 2011	Surveillance	Hospital and outpatient	1,804	1–59	PCR	Naso-/oro-pharyngeal swab
Okiro 2012 (47)	Kenya	May 2002 to Apr 2004	Cross-sectional + surveillance	Outpatient	2,143	0–59	Immuno-fluorescence	Nasal wash
Emukule 2014 (28)	Kenya	Aug 2009 to Jul 2012	Surveillance	Hospital and outpatient	2,169	0–59	PCR	Naso-/oro-pharyngeal swab
Buchwald 2020 (24)	Mali	Oct 2012 to May 2013	Prospective cohort study	Community (RCT)	1,871	0–6	PCR	NR
Dearden 2018 (27)	South Africa	Jan 2013 to Dec 2016	Chart review	Hospital and outpatient	1,127	0–24	Immuno-fluorescence (84%)	Nasopharyngeal aspirate

Study	Country	Years	Study design	Setting	N	Cohort age (mo)	RSV test	Specimen
Zar 2020 (59)	South Africa	Mar 2012 to Mar 2015	Surveillance	Community	1,143	0–24	PCR	Nasopharyngeal swab
Kyeyagalire 2014 (43)	South Africa	Jan 2007 to Dec 2012	Modeling study using surveillance data	Hospital (private)	530,345	All ages	NR	NR
AMRO								
Atwell 2016 (21)	Argentina	May 2013 to Oct 2013	Retrospective cohort study	Hospital	1,591	0–23	PCR	Nasal aspirate
Ferolla 2013 (29)	Argentina	RSV season 2011	Prospective cohort study	Hospital and community	1,293	0–23	PCR	Nasal aspirate
Geoghegan 2017 (33)	Argentina	RSV seasons 2011 to 2013	Prospective cohort study	Hospital and community	3,947	0–11	PCR	Nasopharyngeal wash
Luchsinger 2014 (44)	Chile	RSV seasons 2010 to 2011	Cross-sectional	Hospital and outpatient	124	0–5	Immuno-fluorescence or PCR	Nasopharyngeal swab

Study	Country	Years	Study design	Setting	N	Cohort age (mo)	RSV test	Specimen
Fischer Langley 2013 (30)	Guatemala	Nov 2007 to Jul 2010	Surveillance	Hospital	2,193	0–59	PCR	Naso-/oro-pharyngeal swab
McCracken 2013 (45)	Guatemala	Nov 2007 to Dec 2012	Surveillance	Hospital and outpatient	8,843	All ages	PCR	Naso/oro-pharyngeal swab
Vizcarra-Ugalde 2016 (56)	Mexico	May 2003 to Dec 2014	Cross-sectional	Hospital	3,822	0–59	Immuno-fluorescence or PCR	NR
Kubale 2020 (42)	Nicaragua	Sep 2011 to Sep 2016	Prospective cohort study	Community	833	0–23	PCR	Naso-/oro-pharyngeal swab
Wu 2015 (57)	Peru	Mar 2009 to Sep 2011	Surveillance	Community	892	0–35	PCR	Nasal swab
EMRO								
Freeman 2020 (32)	Jordan	Mar 2010 to Dec 2012	Active surveillance	Hospital	1,271	0–23	PCR	Nasopharyngeal swab
Khuri-Bulos 2018 (40)	Jordan	Mar 2010 to Mar 2013	Surveillance	Hospital	3,168	0–23	PCR	Naso-/oro-pharyngeal swab

Study	Country	Years	Study design	Setting	N	Cohort age (mo)	RSV test	Specimen
Yanis 2021 (58)	Jordan	Mar 2010 to Mar 2013	Active surveillance	Hospital	3,168	0–23	PCR	Naso-/oro-pharyngeal swab
Assaf-Casals 2015 (18)	Lebanon	Oct 2012 to Mar 2014	Retrospective and prospective cohort study	Hospital	443	0–13 years	Antigen test	Nasopharyngeal wash
Ali 2017 (20)	Pakistan	Aug 2009 to Jul 2012	Prospective cohort study	Hospital	1,150	0–59	PCR	Throat swab
EURO								
Kadmon 2020 (36)	Israel	RSV seasons 2012 to 2016	Retrospective chart review	ICU	276	0–24	PCR or antigen test	NR
Kassem 2019 (38)	Israel	Jan 2008 to Dec 20111	Cross-sectional study	Hospital	369	0–23	Chromato-graphic immune-assay	Nasopharyngeal swabs or aspirates
Alan 2016 (19)	Turkey	Oct 2013 to Jan 2014	Prospective cohort study	Hospital	1,277	0–5	Antigen test	NR

Study	Country	Years	Study design	Setting	N	Cohort age (mo)	RSV test	Specimen
Hacımustafaoglu 2013 (35)	Turkey	Mar 2010 to Feb 2011	Cross-sectional	Hospital	671	0–24	Antigen test	Nasal swab
Turkish Neonatal Society 2012 (52)	Turkey	May 2008 to Sep 2010	Prospective cohort study	Hospital	3,464	0–23	Antigen test	Nasal wash, nasopharyngeal aspirates, or nasal/naso-pharyngeal swabs
SEARO								
Kalane 2022 (37)	India	Jul 2021 to Sep 2021	Retrospective cohort study	Hospital (NICU)	16	0–1	PCR	Pharyngeal swab
Simões 2021 (51)	India	Sep 2013 to Mar 2020	Surveillance	Hospital and community	505	0–23	PCR	Nasopharyngeal swab
Chu 2016 (25)	Nepal	Apr 2011 to May 2014	Prospective cohort study	Community (RCT)	3,509	0–6	PCR	Nasal swab
WPRO								
Teck 2019 (54)	Malaysia	NR	Cross-sectional study	Hospital	412	1–24	Antigen test	Nasopharyngeal wash

Study	Country	Years	Study design	Setting	N	Cohort age (mo)	RSV test	Specimen
Tam 2020 (53)	Singapore	Jan 2005 to Dec 2014	Modeling study using primary care and hospital data	Hospital and outpatient	18,323	0–29	Immuno-fluorescence	NR
Ueno 2019 (55)	Philippines	Apr 2014 to Mar 2016	Prospective cohort study	Community	3,817	0–59	PCR	Nasopharyngeal swabs
**Studies focusing on premature infants**								
AMRO								
Benítez-Guerra 2020 (22)	Mexico	Jan 2014 to Dec 2019	Prospective cohort study	Hospital	44	0–12	PCR	Respiratory samples
Ochoa 2014 (46)	Peru	Mar 2009 to Mar 2010	Prospective cohort study	Hospital	211	0–12	Immuno-fluorescence	Nasopharyngeal swab
EURO								
Greenberg 2014 (34)	Israel	Nov to Mar seasons	Prospective cohort study	Hospital	1,058	0–23	Immuno-fluorescence	Nasopharyngeal wash

Study	Country	Years	Study design	Setting	N	Cohort age (mo)	RSV test	Specimen
		2011 to 2014						
Ozkan 2021 (48)	Turkey	Oct 2015 to Mar 2017	Prospective cohort study	Hospital (ER)	307	0–24	Antigen test	Nasopharyngeal wash
SEARO								
Satav 2021 (50)	India	Sep 2016 to Mar 2020	Surveillance	Hospital and community	7,110 (1,113 pre-term)	0–24	PCR	Nasopharyngeal swab
**Postmortem studies**								
AFRO								
Blau 2021[a] (23)	Multi-country[a]	Dec 2016 to Dec 2019	Mortality surveillance	Health facilities	1,213	0–59	PCR	Lung tissue/ nasopharyngeal swab
Forman 2021 (31)	Zambia	Aug 2017 to Dec 2019	Retrospective cohort study	Hospital	720	0–5	PCR	Nasopharyngeal swab
Cohen 2018 (26)	South Africa	Jan 2009 to Dec 2013	Modeling study using	Hospital and community	41,548	0–59	NR	NR

Study	Country	Years	Study design	Setting	N	Cohort age (mo)	RSV test	Specimen
			surveillance data					
EMRO								
Kazi 2021 (39)	Pakistan	Aug 2018 to Mar 2020	Surveillance	Hospital and community	377	0–5	PCR	Nasopharyngeal swab

AFRO, African Region; AMRO, Region of the Americas; EMRO, Eastern Mediterranean Region; ER, emergency room; EURO, European Region; ICU, intensive-care unit; mo, months; NR, not reported; NICU, neonatal intensive-care unit PCR, polymerase chain reaction; RCT, randomized controlled trial; RSV, respiratory syncytial virus; SEARO, Southeast Asia Region; WPRO, Western Pacific Region.

[a]Ethiopia, Kenya, Mali, Mozambique, Sierra Leone, and South Africa. Bangladesh (in SEARO) was also included, but no deaths due to RSV were recorded.
